# Curcumin and Cancer

**DOI:** 10.3390/nu11102376

**Published:** 2019-10-05

**Authors:** Antonio Giordano, Giuseppina Tommonaro

**Affiliations:** 1 Sbarro Institute for Cancer Research and Molecular Medicine and Center of Biotechnology, College of Science and Technology, Temple University, BioLife Science Bldg, Suite 431-1900 N 12th Street, Philadelphia, PA 19122, USA; giordano@temple.edu; 2 Institute of Biomolecular Chemistry, National Research Council of Italy, Via Campi Flegrei, 34-80078 Pozzuoli, Italy

**Keywords:** curcumin, cancer, cell signaling pathways, *Curcuma longa*

## Abstract

Curcumin, a polyphenol extracted from *Curcuma longa* in 1815, has gained attention from scientists worldwide for its biological activities (e.g., antioxidant, anti-inflammatory, antimicrobial, antiviral), among which its anticancer potential has been the most described and still remains under investigation. The present review focuses on the cell signaling pathways involved in cancer development and proliferation, and which are targeted by curcumin. Curcumin has been reported to modulate growth factors, enzymes, transcription factors, kinase, inflammatory cytokines, and proapoptotic (by upregulation) and antiapoptotic (by downregulation) proteins. This polyphenol compound, alone or combined with other agents, could represent an effective drug for cancer therapy.

## 1. Introduction

The most representative polyphenol component extracted from the rhizomes of *Curcuma longa* (known as turmeric) is curcumin. It was isolated for the first time in 1815 by two scientists, Vogel and Pelletier, from Harvard College Laboratory. Since then, the scientific interest towards curcumin has increased and, more and more, its health benefits have been discovered.

Curcumin belongs to a chemical class of polyphenols; it is known as diferuloylmethane and its IUPAC name is (1E,6E)-1,7-bis(4-hydroxy-3-methoxyphenyl)-1,6-heptadiene-3,5-dione, with a chemical formula of C_21_H_20_O_6_ and a molecular weight of 368.38. The chemistry of curcumin is at the basis of its several biological activities.

The therapeutic benefits of curcumin have been demonstrated in multiple chronic diseases: inflammation, arthritis, metabolic syndrome, liver disease, obesity, neurodegenerative diseases and, above all, in several cancers. As a result of a recent bibliographic research, we found 12,595 papers on curcumin (1924–2018) and 4738 (1983–2018) of which were on curcumin and cancer; that means 37% of the published papers on curcumin has cancer as the major targeted disease (https://www.ncbi.nlm.nih.gov/pubmed). However, the abovementioned activities seem to be due mostly to the antioxidant and anti-inflammatory effects of curcumin.

Cancer is one of the primary causes of death in industrialized countries [1]. In recent years, the early diagnosis and increase in therapeutic options has reduced the death rate. However, the growth of drug-resistant cancers necessitates the search for innovative and more effective drugs [2]. It is worth noting that cancer cells are characterized by deregulated signaling pathways involving proliferation, apoptosis, and angiogenesis [3,4].

In this scenario, curcumin represents a promising candidate as an effective anticancer drug to be used alone or in combination with other drugs. It affects different signaling pathways and molecular targets involved in the development of several cancers (Table 1).

The present review collects the most recent studies on the actions of curcumin in the prevention and treatment of different types of cancers.

### Immunomodulatory Effects of Curcumin

Many evidence suggest that the disorder of inflammatory pathways play a key role in cancer development [5].

The inflammation process induces an increased production of pro-inflammatory molecules such as cytokines, ROS, cyclooxygenase (COX-2), transcription factors including nuclear factor κB (NF-κB), protein kinases B (AKT), activator protein 1 (AP1), signal transducer and activator of transcription 3 (STAT3), causing the initiation and development of cancer [6,7].

Curcumin exerts its immunomodulatory ability by interacting with several immune mediators, hence its anticancer property.

Nuclear factor κB is a pro-inflammatory transcription factor that modulates the expression of different proteins—such as cytokines interleukin (IL)-1, IL-2, and interferon-γ (IFNγ)—involved in multiple cell signaling pathways associated with cancer progression and inflammation [8]. Phosphorylated NF-κB binds DNA and starts the transcription of oncogenes which block apoptosis and initiates cellular proliferation and angiogenesis. Curcumin suppress NF-κB activity by inhibiting the phosphorylation by I kappa B kinase (IκB) and impeding nuclear translocation of the NF-κB p65 subunit.

Similarly, the transcriptional factor AP-1 (Activator Protein-1), known to be related to anti-apoptotic, mitogenic, and pro-angiogenic genes, is downregulated by curcumin. Indeed, curcumin is reported to exert anticancer properties in different in vitro models through the inhibition of AP-1 and NF-κB factors [9].

One member of the STAT family, STAT3, is described as a common target for several signaling pathways regulating oncogenes, as well as modulating the transduction of pro-inflammatory cytokines and growth factors [10]. This factor contributes to the growth and survival of the cell, increasing the expression of anti-apoptotic proteins such as Bcl-2 and Bcl-xL, thereby blocking apoptosis. Several factors, such as IL-6, as well as EGFR, PDGF, leukemia inhibitory factor (LIF), oncostatin M, and the ciliary neurotrophic factor (CNTF) family of cytokines, are reported to be STAT3 activators. Moreover, STAT3 is reported to be a molecular target of curcumin in several tumors, both directly and indirectly by inhibition of IL-6 [11,12,13].

In addition to the abovementioned transcriptional factors, pro-inflammatory cytokines—such as tumor necrosis factor alpha (TNF-α) and interleukins—have a notable role both in the inflammatory process and cancer disease. Tumor necrosis factor alpha activates NF-κB, then inflammatory genes (*5-LOX*, *COX-2*), inflammatory cytokines, molecules that adhere to cells, and inducible nitric oxide synthase (iNOS) are expressed. Therefore, the transcription of TNF-α and, thus, the expression of inflammatory genes are blocked by curcumin [9].

The transcriptional factors NF-κB and AP-1 are also regulated by protein kinases (IκB kinases, MAPKs, and ERK1/2), hence their modulation is a strategy in cancer control and prevention. There is some evidence for the ability of curcumin to inhibit protein kinases inducing apoptotic activity [14]. In addition, curcumin exerts its anticancer activity by acting on the level of cyclin D1 that is an important regulator of cell cycle progression and can act as a transcriptional co-regulator. Indeed, high levels of cyclin D1 has been related to the development and progression of cancer. The suppression of cyclin D1 by curcumin occurs through NF-κB inhibition [15].

However, the immunomodulatory property of curcumin is exerted not only towards molecular targets, but also cellular components such as macrophages, dendritic cells, and both T and B lymphocytes [16].

## 2. Breast Cancer

Nowadays, breast cancer is the most widespread malignant tumor among the female adult population. It is the leading cause of death due to the presence of cancer in women around the world [17]. Although the best approach to enhancing breast cancer outcomes and survival remains early detection, the use of different drugs is still an effective treatment for breast cancer. Because more than 70% of breast cancer cases are estrogen receptor (ER) positive type, antiestrogens are often used as the main treatment. However, growing evidence has shown that the combination of different drugs represents the best strategy in breast cancer management.

In the proliferation of breast cancer cells, NF-κB—the proinflammatory transcription factor—plays a key role. It regulates more than 500 different genes and governs the expression of proteins involved in cellular signaling pathways, resulting in the development of cancers and inflammation. Compounds able to interact with NF-κB, by its inhibition, may be used in cancer therapy. Curcumin displayed the ability to affect the breast cancer cell proliferation and invasion by downregulating the NF-κB inducing genes [18,19].

Another target that acts on the proliferation of breast cancer cells is the human epidermal growth factor receptor 2 (HER2), a tyrosine kinase (TK) receptor belonging to EGFR family. The HER2 is considered as a drug target for cancer therapy since its overexpression is involved in the development of many types of cancer [20]. Curcumin, alone or in combination with its analogues, may inhibit breast cancer cell lines though inhibiting of HER2-TK [21]. Its suppressing action towards HER2 was improved in selectivity by immune-liposome encapsulation [22].

The alterations (mutation and amplification) in the protein kinase B, named Akt, are related to carcinogenesis [23]. Together with Akt, mTor (kinase) interfered in the control of cancer cell growth and proliferation [24]. In breast cancer cells, curcumin downregulated Akt protein in a dose- and time-dependent manner, and induced autophagy and suppression of the ubiquitin-proteasome pathway [25]. Moreover, it has been hypothesized that the apoptotic and autophagy abilities of curcumin in breast cancer cells are conducted by blocking the PI3K/Akt signaling pathway [26]. The apoptotic effect of curcumin has also been observed after treatment of MCF-7 cells with curcumin plus PI3K inhibitor, suggesting a synergistic effect [27].

Curcumin is also able to interfere with the cell signaling pathway of EGFR, a family of receptor tyrosine kinases, that is reported to be associated with the proliferation, adhesion, migration, and differentiation of cancer cells [28,29]. Therefore, modulation of EGFR represents a good strategy for cancer therapy. Curcumin inhibited the growth and proliferation of breast cancer cells by reducing EGFR signaling and decreasing EGFR and Akt levels [30,31].

Curcumin exerts its chemo-preventive and antiproliferative capacities by means of modulation of the transcription factor Nrf2, which regulates different genes for proteins responsible for the detoxification of electrophiles and ROS, as well as the elimination or restoration of some of their damaged products [32,33].

Antiproliferative abilities of curcumin are estrogen dependent in ER (estrogen receptor)-positive MCF-7 breast cancer cells. Indeed, it represses the expression of ER in downstream genes such as *pS2* and *TGF-beta* (transforming growth factor) in ER-positive MCF-7 cells, and this capacity is also dependent on the presence of estrogen.

However, curcumin showed effective anti-invasive activities in vitro that are not estrogen dependent in ER-negative MDA-MB-231 breast cancer cells. These interesting activities appeared to be mediated through the downregulation of MMP-2 (matrix metalloproteinase) and the upregulation of TIMP-1 (tissue inhibitor of metalloproteinase), two molecules involved in the regulation of cancer cell invasion [34].

Recently, the potential of curcumin to modulate the expression of miRNAs (non-coding sequences of 18–22 nucleotides involved in several diseases, including cancer) in breast cancer cells has been reported [35]. Curcumin was able to affect the expression of oncogenic (miR-19a and miR-19b) and tumor-suppressive miRNAs (miR-15a, miR-16, miR-34a, miR-146b-5p, and miR-181b) in breast cancer cells. As a consequence, the suppression of tumorigenesis and metastasis, and induction of apoptosis were observed.

## 3. Lung Cancer

Lung cancer is a widespread tumoral disease and it is the major cause of cancer-related mortality in men worldwide [36]. Depending on the stage and the tumor’s aggressiveness, the five-year survival rate in populations with lung cancer varies from 4–17% [37]. Recently, much progress has been made in regards to improving early diagnosis, lung cancer screening, and innovative therapies.

Curcumin exhibited its therapeutic efficiency in lung cancer treatment by means of the downregulation of NF-κB in human lung cancer cell lines A549 and also by acting on the JAK2/STAT3 signaling pathway, inhibiting JAK2 activity [13,38].

Moreover, curcumin inhibited cell proliferation and induced apoptosis of human non-small cell lung cancer cells via the upregulation of microRNA-192-5p and suppression of the PI3K/Akt signaling pathway [39].

In lung tumor proliferation, neutrophil elastase (an important regulator of inflammatory processes) and α1-antitrypsin (natural inhibitor of neutrophil elastase) play prominent roles in the inflammation mechanism and curcumin repressed neutrophil elastase-induced tumor proliferation via upregulating α1-antitrypsin expression in vitro and in vivo [40]. However, it has been reported that a novel catanionic lipid nanosystem (CLN) incorporating curcumin (CCM) exhibited better cytotoxicity in Lewis lung cancer (LLC) cells, increasing antiproliferative, proapoptotic, and anti-invasive activities and induction of cell cycle arrest. The novel nanosystem caused apoptosis in LLC cells by CCM through the PI3K/Akt/FoxO1/Bim cellular target [41].

Curcumin also exhibited its proapoptotic activity in lung adenocarcinoma cells by suppressing expression of *COX-2*, EGFR, and extracellular signal-regulated kinase (ERK) 1/2 activities, which correlated with elevated apoptosis and reduced survival of lung adenocarcinoma cells [42]. Another study discussed the consequences of curcumin treatment on erlotinib-resistant NSCLC cells. Erlotinib was an EGFR-tyrosine kinase inhibitor (EGFR-TKI) and the combined treatment of curcumin and erlotinib remarkably reduced tumor growth of erlotinib-resistant NSCLC cells in vivo [43]. The Wnt family of signaling proteins are involved in different developmental events during embryogenesis and they are also implicated in adult tissue homeostasis. The dysregulation of this signaling pathway is often associated with several diseases, in particular cancer [44]. Curcumin was able to influence the cellular progression in non-small cell lung cancer and induced G0/G1 phase arrest through MTA1 (metastasis-associated protein 1)-mediated inactivation of Wnt/β-catenin pathway [45]. An interesting study reported the potential of curcumin in regulating the expression of miRNA, a portion of the human genome sequence for non-coding sequences involved in different diseases, in particular, cancers. In lung carcinoma cells, a reduction of miRNA-186 expression was detected after treatment with curcumin [46]. The in vivo studies were accomplished in transgenic mice carrying the pulmonary tumor producing human vascular endothelial growth factor A165 (hVEGF-A165). In Clara cells of the lungs of transgenic mice, curcumin specifically suppressed hVEGF-A_165_ overexpression to normal. Additionally, a reduction of Cyclin A and Cyclin B (proteins involved in the S to M phase transition) was detected [47].

## 4. Hematological Cancers

Hematological tumors include different group of cancers that affect the blood, bone marrow, and lymphatic systems. The most widespread categories are lymphoma, leukemia, and multiple myeloma [48].

Leukemia is a cancer concerning the blood or bone marrow characterized by an anomalous proliferation of blood cells. Curcumin has been found to suppress TNF-α-induced nuclear translocation and DNA binding of NF-κB through suppression of IκBα phosphorylation and degradation in the human myeloid ML-1a cells [49]. Moreover, curcumin exhibited apoptosis in B-cell chronic lymphocytic leukemia (CLL-B) via downregulation of STAT3, AKT, NF-κB, and X-linked inhibitor of apoptosis protein (XIAP). It also upregulated the proapoptotic protein BIM [50,51].

The Wilms tumor 1 (*WT1*) gene acts both as an oncogene and as a tumor suppressor. It is involved in the proliferation and vitality of different cancer cells. It was found to be highly expressed in many leukemic cell lines and in patients with acute myeloid leukemia. Curcumin inhibited cell proliferation and clonogenicity in the K562 cell line which expresses *WT1* at a high level (mRNA and protein)—depending on time and dose—through inhibition of the WT1 protein. It also caused cell cycle arrest at the G2/M phase [52]. The *WT1* expression inhibition exerted by curcumin was also reported in patient leukemic cells [53]. In addition, another study reported that curcumin decreased *WT1* gene expression in both transcriptional and translational levels [54].

Curcumin also induced apoptosis through the activation of the JNK/ERK/AP1 pathways in human acute monocytic leukemia THP-1 cells [55].

In chronic myelogenous leukemia (CML) cells, the anticancer properties of curcumin were exerted by upregulating PTEN, one of the mutated or silenced tumor suppressors in human cancer, which is a target of miR-21, a microRNA overexpressed in several cancers. Curcumin induced miR-21-mediated modulation of the PTEN/AKT pathway causing the inhibition of leukemic cell growth, in vitro and in vivo [56].

Lymphomas represent the fifth most common cancer and fifth primary cause of cancer mortality in Western countries. Among them, lymphomas derived from B cells represent more than 80% of diagnosed cases. These kinds of cancers represent clonal proliferations of lymphocytes that are mainly organized according to their maturity (peripheral or mature versus precursor) and lineage (B cell, T cell, and natural killer cell). The pathogenetic mechanisms involved in the development of lymphoma define the classification of lymphoma and the subsequent clinical management of patients [57,58].

In human Burkitt’s lymphoma—a high-grade non-Hodgkin’s lymphoma (NHL)—curcumin affected, by inhibition, the constitutive and radiation-induced expression of the PI3K/AKT pathway and its downstream regulator NF-κB. This effect gave rise to apoptosis in three human Burkitt’s lymphoma cell lines (i.e., Namalwa, Ramos, and Raji) that were treated with ionizing radiation. Hence, curcumin could play an important role in radiotherapy of high-grade NHL by means of inhibition of the PI3K/AKT-dependent NF-κB pathway [59]. The in vivo anticancer effects of curcumin on human Burkitt’s lymphoma Raji cells has been reported in a xenograft mouse model through the downregulation of oncogene *c-Myc* and the upregulation of apoptotic proteins [60].

It is reported that curcumin also affected interleukin-1 (IL-1α and IL-1β), a prototypic, potent, multifunctional proinflammatory cytokine involved in tumor progression via expression of metastatic, angiogenic genes, and growth factors. Curcumin reduced carcinogenesis by downregulating proinflammatory cytokine interleukin-1 (IL-1α and IL-1β) via modulation of AP-1 and NF-IL6, respectively, in lymphoma bearing mice [61].

Curcumin also repressed the growth of B lymphoma cells through suppression of the *egr-1* gene (known as nerve growth factor-induced protein A) expression, which affected the suppression of the *c-myc* gene and the anti-apoptotic protein bcl-XL in BKS-2 cells (immature B cell lymphoma) [62].

Multiple myeloma (MM) is a systemic malignant disease of the blood—in most cases fatal— characterized by the uncontrolled proliferation of monoclonal plasma cells in the bone marrow, leading to the production of non-functional intact immunoglobulins or immunoglobulin chains. Curcumin exerted its anticancer potential in multiple myeloma by acting on NF-κB and STAT3 cell signaling pathways. Indeed, curcumin exhibited anticancer potency by means of suppression of IκB kinase and its oral administration was reported to suppress NF-κB in PBMCs (peripheral blood mononuclear cells) from multiple-myeloma patients [49,63].

Moreover, curcumin was able to inhibit IL-6-induced STAT3 phosphorylation and consequent STAT3 nuclear translocation playing a key role in the suppression of MM proliferation [64]. In vitro cultured U266 cells, curcumin was tested in combination with carfilzomib (CFZ, a cytotoxic second-generation proteasome inhibitor). Results showed that curcumin significantly ameliorated in vitro cytotoxic of CFZ on U266 cells, enhanced CFZ induction of p53/p21 axis and apoptosis, deeply reduced NF-κB nuclear accumulation in U266 cells. Then, curcumin may improve the therapeutic efficacy of CFZ, and supply a mechanistic understanding of the antitumor effects of these drug combinations involving activation of the p53–p21 axis and NF-κB inhibition [65].

In a recent paper, curcumin was reported to significantly inhibit the proliferation of MM cells, inducing apoptosis, in a time- and concentration-dependent manner, through the inhibition of the expression of EZH2 in RPMI8226 and U266 cell lines. Curcumin upregulated miR-101 and, subsequently, a lower expression of EZH2 was observed. On the contrary, the expression of EZH2 induced lower expression of miR-101. The results showed the effect and mechanism of curcumin on multiple myeloma via EZH2–miR-101 regulatory feedback loop [66].

## 5. Cancer of Digestive System

### 5.1. Gastric Cancer

Gastric cancer is one of the prominent causes of mortality worldwide in men and women. It is often diagnosed in the final stages because of the absence of symptoms in early stages of development [67,68].

Many studies reported the pharmacological efficiency of curcumin in the treatment of gastric cancer. Curcumin exerted its antitumor action by means of inhibition of antiapoptotic proteins of the Bcl-2 family and elevated the expression of p53, Bax, procaspases 3, 8, and 9 [69].

Curcumin caused dissipation of mitochondrial membrane potential (MMP) and the release of cytochrome c into the cytosol of SGC-7901 cells eliciting apoptosis. Moreover, the downregulation of Bcl-2 and upregulation of Bax that provoked the cleavage of caspase-3 and increased cleaved PARP was also reported [70].

The strong antioxidant activity exhibited by curcumin by inhibition of ROS also contributed to cancer chemoprevention [71].

The STAT3 pathway has been reported as another target of curcumin. Indeed, curcumin downregulated pSTAT3 levels, survivin expression, and gastric cancer cell viability in a dose-dependent manner. Besides, 5-fluorouracil in combination with curcumin showed a synergistic effect of survivin and STAT3 levels resulting in enhanced cell death in gastric cancer cells [11].

It has been reported that curcumin significantly decreased the expression of cyclin D and inhibited p21-activated kinase1 (PAK1) activity giving rise to the suppression of proliferation and invasion of gastric cancer cells [72]. Really, it acted on cell cycle arrest at the G2/M phase in AGS cells via decreasing cyclin D1 and increasing cyclin B1 in a dose-dependent manner [73].

In addition, curcumin has been reported to act on caspase-3 (mediator of apoptosis) by activation and on the Akt/mTOR/p70S6 signal pathway by inhibition [74,75].

### 5.2. Colorectal Cancer

Colorectal cancer is one of the most widespread cancers, affecting men and women equally. Because of its malignant features, patients rarely heal, and recurrence is common. In colorectal cancer, curcumin exhibited its therapeutic action by affecting several cell signaling pathways.

Curcumin inhibited DMH (1,2-Dimethylhydrazine)-induced rat colorectal carcinogenesis and the growth of the in vitro cultured HT 29 cell line by suppressing the PPARγ signal transduction pathway [76]. In addition, curcumin also suppressed the expression of cyclooxygenase-2 (COX-2), p53, and pre-mRNA processing factor 4B (Prp4B) [77,78].

The AMP-activated protein kinase (AMPK) pathway has gained more interest as an important pathway involved in cancer control. Curcumin has been reported as an inhibitor of colorectal cancer invasion by means of AMPK-induced inhibition of NF-κB, urokinase-type plasminogen activator (uPA) activator, and matrix metalloproteinase-9 (MMP9) [79].

Curcumin has been reported as an agent able to prevent colorectal cancer proliferation by blocking the cell cycle and accelerating apoptosis. It exerted this action affecting thymidylate synthase and its transcription factor E2F-1. This effect caused cell cycle inhibition via downregulation of NF-κB and other survival pathways [80]. Besides, curcumin downregulated the kinase CDK2, leading to the G1 cell cycle [81].

In human colon cancer cells, curcumin significantly inhibited cell growth. Further, it also inducted apoptosis through a mitochondria-mediated pathway. Curcumin induced the release of cytochrome c, significantly increased Bax and p53, and showed a marked reduction of Bcl-2 and survivin in LoVo cells [82].

In human colorectal cancer HCT116 and HT29 cells, curcumin downregulated the expression and activity of hexokinase II (HKII) in a concentration-dependent manner and induced dissociation of HKII from mitochondria, resulting in mitochondrial-mediated apoptosis [83].

Additionally, in colon cancer cells SW480, curcumin targeted the WNT/catechin pathway through a decrease of miR-130a expression and exerting its anti-tumor activity by inhibition of cell proliferation rather than promoting cell apoptosis [84]. Moreover, curcumin also targeted the miR-491/PEG10 pathway, thus inhibiting proliferation and inducing apoptosis of colon cancer cells [85].

### 5.3. Pancreatic and Hepatic Cancers

Pancreatic cancer is a very fatal type of cancer with a one-year survival rate of only 10–28% and a five-year survival rate of around 7% [86,87]. Mutations in oncogenes and tumor suppressor genes as well as alterations of different signaling pathways are involved in the initiation, promotion, and progression of pancreatic cancer.

Curcumin has been shown to have an effect on pancreatic cancer cells’ vitality, in vitro and in vivo, by means of inhibition of NF-κB, COX-2, CD-31, VEGF, and IL-8 [88,89]. In addition, curcumin treatment also inhibited STAT3 activation in patients with pancreatic cancer [90].

In pancreatic cancer cells, curcumin has been reported to induce FoxO1 expression in pancreatic cancer cells by acting on PI3K/Akt signaling, which caused cell cycle arrest and apoptosis induction [91]. Moreover, curcumin induced apoptosis by inhibition of PI3K/Akt signaling and upregulation of PTEN [92].

Additionally, curcumin inhibited RelA–DNA binding, suppressed COX-2, EGFR, extracellular signal-regulated kinase (ERK1/2), and Notch signaling, effecting elevated apoptosis and reduced survival of pancreatic adenocarcinoma cells [42,93]. Moreover, curcumin downregulated miRNA-199a and upregulated miRNA-22 including target genes *SP1* and *ESR1* [94].

In pancreatic cancer, cells often express Wilms tumor gene 1 (*WT1*). Curcumin treatment influenced the expression of *WT1* on mRNA, which resulted in significant downregulation. In addition, co-treatment of curcumin with siRNA (small inhibitory RNA) targeting *WT1* triggered an increase in the inhibition of cell proliferation compared to curcumin-treated cells alone [95].

Hepatic cancer is one of the most common cancers with dismal prognosis and is the third highest cause of cancer mortality worldwide [96].

Curcumin induced DNA damage to both the mitochondrial and nuclear genomes in human hepatoma G2 cells. The study showed that low levels of curcumin did not induce DNA damage but acted as an antioxidant agent in carcinogenesis. At high doses, curcumin imposed oxidative stress by increasing ROS generation and lipid peroxidation and damaged DNA curcumin [97]. The treatment of hepatoma cells with curcumin led to an increase of ROS that effected the histone acetyltransferase (HAT), an enzyme controlling the state of histone acetylation in vivo. In particular, the exposure of human hepatoma cells to curcumin caused a significant decrease of histone acetylation by acting on ROS generation [98].

The Notch-1 signaling pathway is also targeted by curcumin. Indeed, it was reported that curcumin damaged Notch-1 signaling within the Notch intracellular domain in the HEP3B, SK-Hep-1, and SNU449 cell lines. Moreover, curcumin exhibited protection against diethylnitrosamine (DENA)-induced hyperplasia and HCC in rodents by decreasing expression of p21-Ras, P53, and NF-κB [99].

In BALB/c mice treated with N-bis(2-hydroxypropyl) nitrosamine (DHPN), curcumin inhibited liver adenoma formation and growth by enhancing the lipid peroxidation and antioxidant liver enzymes [100].

In the highly invasive SK-Hep-1 cell line of human hepatocellular carcinoma, curcumin significantly inhibited cellular migration and invasion of SK-Hep-1. The effect of curcumin was related to its inhibitory action on MMP-9 (matrix metalloproteinase) secretion [101].

## 6. Other Cancers

The second most common type of cancer diagnosed in men is prostate cancer. In prostate cancer, curcumin exhibited its therapeutic effects by modulating multiple cell signaling pathways.

In human androgen-independent (DU145) and androgen-dependent (LNCaP) prostate cancer cell lines, curcumin decreased the expression of antiapoptotic genes *Bcl2* and *Bcl-xL*, and induced procaspase-3 and procaspase-8 leading to apoptosis. Treatment of cells with curcumin inhibited both constitutive (DU145) and inducible (LNCaP) NF-κB activation, and potentiated TNF-induced apoptosis [102].

Curcumin has been reported to abolish CAF (cancer-associated fibroblast)-induced invasion and EMT (epithelial–mesenchymal transition), and inhibited ROS production and CXCR4 and IL-6 receptor expression through inhibiting MAOA/mTOR/HIF-1α (monoamine oxidase A/mammalian target of rapamycin/hypoxia-inducible factor-1α) signaling, thereby supporting the therapeutic effect of curcumin in prostate cancer [103].

In castration-resistant prostate cancer (CRPC) cells, the mixed treatment of curcumin with docetaxel (DTX) and nelfinavir (NFR) caused significant suppression of phosphorylated-Akt and induction in phosphorylated-eIF2α, which means an induction of ER stress leading to apoptosis. This study showed the ability of curcumin to chemo-sensitize the CRPC cells to DTX therapy [104].

The combination of curcumin with β-phenylethyl isothiocyanate also affected the proliferation of human prostate cancer PC-3 cells by inhibiting another signaling pathway, EGFR. Its inhibition led to the inhibition of proliferation as well as the programmed death of prostate cancer cells [105].

Another study reported the antiproliferative potential of curcumin against prostate cancer cells through modulation of the Nrf2 pathway [106] and nuclear β-catenin transcription activity, as well as membrane β-catenin levels [107].

Curcumin also affected the prostate-specific antigen (PSA) by reducing the expression of AP-1, cyclin D1, NF-κB, and cAMP response element-binding (CREB). In addition, curcumin has been reported to inhibit androgen-receptor-dependent NKX3.1 expression by means of the modulation of androgen receptors [108,109].

A recent study performed with human prostate cancer stem cells (HuPCaSCs) treated with curcumin, reported the inhibition of in vitro proliferation and invasion as well as cell cycle arrest as a consequence of the miR-145 overexpression in curcumin-treated HuPCaSCs [110].

However, curcumin also affected the expression of the miR-143/miR-145 cluster. Indeed, in human prostate cancer cell lines LNCaP, DU145, and PC3, the curcumin pretreatment and the miR-143 overexpression increased radiation-induced cancer cell growth inhibition and apoptosis. In summary, curcumin could sensitize prostate cancer cells to radiation both by miR-143 activation and miR-143-mediated autophagy inhibition [111].

The anticancer effect of curcumin, both alone and/or in combination with other compounds, has also been reported in brain tumors. Bojko et al. (2015) [112] reported curcumin as a potent adjuvant agent in the treatment of human brain cancer involving selective EGFR kinase inhibitors such as tyrphostins AG494 and AG1478. Indeed, curcumin increased the cytostatic and/or cytotoxic potential of AG494 and AG1478, and decreases in viability, stimulation of apoptotic processes, irreversible DNA damage, and ROS were observed in the culture of glioblastoma cells treated with a mixture of curcumin and tyrphostins [113].

Additionally, curcumin interfered with the PI3K/Akt and NF-κB signaling pathways by activation, downregulated *Bcl-xL*, and induced the mitochondrial dysfunction together with caspase-3 activation [114]. Besides, curcumin upregulated p53 expression, followed by induction of p21 WAF-1/CIP-1 and ING4 (inhibitor of growth, family 4) [115].

In bone fibrosarcoma cancer cells, curcumin also showed antitumor potential by interfering with multiple cellular targets.

Curcumin induced apoptosis in fibrosarcoma cells by means of the downregulation of NF-κB, IL-6, and IL-11, hence altering the modulatory effects of TGF-b (transforming growth factor beta). Furthermore, curcumin inhibited Erk and Bcl2 expression, suppressed MMP-13 expression, and induced apoptosis in bone cancer cell lines [115,116].

Head and neck cancers, most of which are squamous cell carcinomas (HNSCC), include cancers of the oral cavity, pharynx, and larynx, and their incidence increases with high consumption of tobacco and alcohol [117,118]. Many in vitro and in vivo studies report the efficiency of curcumin in the management of head and neck cancers. In vitro studies performed on HNSCC cell lines described their apoptosis after treatment with curcumin. Curcumin induced apoptosis by direct (pro-apoptotic and anti-apoptotic gene expression) and indirect mechanisms (cell cycle arrest in G2/M phase and increasing of sub-G1 cell population), by acting on different cellular targets (NF-κB, cyclin D1, Bcl-2, NOS, COX-2, interleukins, TNF-a, and MMP-9) [119,120]. Combination chemotherapy using curcumin together with other anticancer drugs (5-FU, cisplatin, doxorubicin) has been described as a good strategy to improve the therapeutic approach in head and neck cancer management. Furthermore, innovative systems (including nanoparticles, micelles, liposomes, and hydrogel) for drug delivery could be adopted in the treatment of head and neck cancers [121,122].

Malignant mesothelioma (MM) is a primary tumor—usually involving pleural and peritoneal spaces—which is extremely aggressive and lethal with scarce healing and successful treatment. Asbestos exposure is the main cause of tumor development, but also radiation exposure and genetic factors could give rise to malignant mesothelioma.

Because MM cancer cells exhibit multiple abnormal modulations of signaling cellular pathways, a multifunctional drug like curcumin seems to be very efficient in MM treatment. In vitro studies reported the anticarcinogenic properties of curcumin through the induction of pyroptosis through activation of caspase-1 and increased release of high-mobility group box 1 (HMGB1). Moreover, curcumin also downregulated levels of inflammasome-related gene expression involved in inflammation, e.g., NF-κB, toll-like receptors (TLR), and IL-1β [123]. Curcumin has been reported to induce autophagy in the ACC-MESO-1 human malignant pleural mesothelioma cell line increasing LC3B-II/LC3B-I expression and inducing the formation of autophagosomes [124]. In other in vitro models using human (H2373, H2452, H2461, and H226) and murine (AB12) malignant pleural mesothelioma cells, curcumin increased levels of proapoptotic proteins (Bax), stimulated PARP cleavage, and induced apoptosis. Apoptosis induction was also observed in vivo after oral administration of curcumin at a dose of 500 mg/kg [125].

## 7. Bioavailability of Curcumin

Despite the good prospective for curcumin in the management of cancer diseases, its clinical development is limited because of its scarce bioavailability and low aqueous solubility. In clinical trials, it was reported that curcumin given orally at a dose of 8 g/day in humans, a rapid transformation into metabolites occurred resulting in a low level of free curcumin in plasma (<2.5 ng/mL) [126].

Many efforts have been made to improve the stability, solubility and, most of all, the bioavailability of curcumin. An adopted strategy has been the achievement of derivatives of curcumin by chemical modifications or chemical synthesis of its analogues. Since the key sites of the molecule for the anti-tumor activity seemed to be the oxyphenyl and carbon chain moieties, many studies have focused on chemically changing the abovementioned key sites, obtaining very promising results [127]. Though curcumin analogues represent a good approach to improving the bioavailability of curcumin, many studies have been directed towards the development of innovative delivery systems for improving the pharmacokinetics of curcumin. Curcumin encapsulated in protein nanoparticles exhibited a better anticancer activity, detectable by the loss of MCF-7 cells’ viability and an enhanced oral bioavailability in rats [128].

Two promising nanocurcumin formulations, Lipocurc™ (liposomal curcumin for infusion) and Meriva^®^, have been shown to increase the bioavailability of curcumin and lead to better treatment outcomes in pancreatic and lymphocytic leukemia patients, respectively [129,130,131].

Relevant results have been achieved after oral administration of exosomal curcumin (ExoCUR, a nanoformulation with curcumin mixed with exosomes from bovine milk) in Sprague–Dawley rats. The ExoCur improved curcumin bioavailability as well as antiproliferative activity in multiple cancer cell line models including breast, lung, and cervical cancer compared with the free curcumin, as well as in in vivo for nude mice bearing the cervical CaSki tumor xenograft [132]. It is worth noting the study by Antony et al. (2008), in which the patented formulation, BCM-95^®^ CG (a mix of reconstituting curcumin with the non-curcuminoid components of turmeric) was tested on a human group of volunteers with the aim of estimating the bioavailability of curcumin in blood. The increase in relative bioavailability of BCM-95^®^ CG (BiocurcumaxTM) was about 6.93 fold compared to free curcumin and about 6.3 fold compared to a curcumin–lecithin–piperine formula [133].

Although curcumin has shown poor bioavailability, due also to the fact of its chemical instability, many in vivo studies, particularly preclinical studies, still focus on the therapeutic effects of curcumin, though more large-scale trials including placebos are required for a deep evaluation of its effects on humans.

### Recent Advances

Clinical use of curcumin is still under investigation, both as a monotherapy and in combination with other drugs. In a phase I clinical trial, curcumin was used alone in 15 colorectal cancer patients as an oral formulation. The authors reported the absence of toxicity, the development of significant diarrhea in two patients, and two patients showed stable disease after two months of curcumin treatment [134]. An additional monotherapy clinical trial (phase II) of curcumin as an oral formulation was performed in 25 advanced pancreatic cancer patients. Despite the low levels of curcumin present in plasma (22–41 ng/mL) two patients showed clinical biological activity. Indeed, in one patient a stable disease for >18 months was observed and in another patient, a brief but marked, tumor regression (73%) was ascertained [90]. The assessment of curcumin mixed with other drugs as chemotherapeutic or adjuvant to the standard treatments in cancer disease has been reported. The therapeutic effect of a combination of curcumin with imatinib (tyrosine kinase inhibitor) has been evaluated in 50 chronic myeloid leukemia patients. The mixed treatment was more effective than imatinib alone, although additional studies are needed to confirm the efficacy of the experimental combination [135]. Furthermore, a combination of curcumin with anti-EGFR monoclonal antibodies in pretreated cSCC (cutaneous squamous cell carcinoma) patients has been described as a highly effective strategy in disease control [136].

It is worth noting in the present review some of the most recent clinical trials on curcumin as a therapeutic agent in various cancers (Table 2) (https://clinicaltrials.gov/).

In breast cancer patients, curcumin is under investigation in monotherapy (NCT03980509) and in combination with paclitaxel (NCT03072992). However, the aim of these clinical studies is to evaluate the therapeutic effect of curcumin on the development of primary and metastatic breast cancer as well as to estimate the risk of adverse events.

The use of curcumin against placebo in low-risk, localized prostate cancer patients is evaluated with the aim to reduce cancer progression (NCT03769766).

The safety and tolerability of curcumin mixed with a drug called Lovaza (made with fish oils), which reduces the size of lung nodules, is the subject of a phase II clinical trial (NCT03598309). In another phase II clinical study, curcumin is under evaluation as a adjuvant (food supplement) in patients with advanced and/or refractory cervical cancer, endometrial carcinoma or uterine sarcoma treated with an immunomodulatory cocktail (Vitamin D, aspirin, cyclophosphamide and lansoprazole), followed by pembrolizumab, combined with radiation (NCT03192059).

## 8. Conclusions

**Figure 1:**
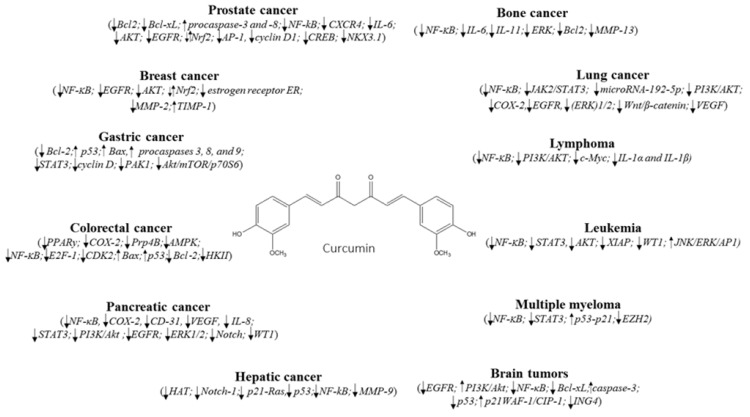
Cell signaling pathways targeted by curcumin in different cancers. ↓ Downregulation/inhibition; ↑ Upregulation/activation; ⇵ Modulation.

The search for new effective drugs able to combat cancer diseases still represents a challenge for many scientists. Natural organisms (e.g., plants, bacteria, fungi) provide many active molecules with a potential application in medicine for the management of many diseases (neurodegenerative, cardiovascular, inflammation, cancers). Curcumin, a polyphenol extracted from the rhizomes of *Curcuma longa,* belong to the most promising group of bioactive natural compounds, especially in the treatment of several cancer types. As reported in the present review, curcumin exhibits anticancer ability by targeting different cell signaling pathways including growth factors, cytokines, transcription factors, and genes modulating cellular proliferation and apoptosis (Figure 1). However, curcumin is not immune from side effects, such as nausea, diarrhea, headache, and yellow stool. Moreover, it showed poor bioavailability due to the fact of low absorption, rapid metabolism, and systemic elimination that limit its efficacy in diseases treatment. Further studies and clinical trials in humans are needed to validate curcumin as an effective anticancer agent.

## Figures and Tables

**Table 1:** Main molecular targets of curcumin.

Molecular Targets of Curcumin						
Transcription Factors	Growth Factors	Inflammatory Cytokines	Apoptotic Proteins	Protein Kinases	Receptors	Cell Survival/ Proliferative Proteins
*ERG-1*, ERE, STAT-1, STAT-3, STAT-4, STAT-5, Notch-1, NF-κB, PPAR-γ, WTG-1, β-catechin	FGF, VEGF, TGF-β1, TF, CTGF, EGF	Prostaglandine, TNF, IFN, interleukins, COX-2, MCP-1, MaIP	Cytochrome c, PARP, Bax, Caspase-3, Caspase-8, Caspase-6, Caspase 10, FADD	MAPK, EGFR, ERK, IL-1 RAK, PKA/B/C, Bcr-Abl, JNK, IKK	HER-2, CXCR4, EGFR, H2R, IL-8R, LDL-R, ITPR	Survivin, Mcl-1, Bcl-xL, cIAP-1, cIAP-2, Bcl-2, cMyc, PCNA, cyclin D1

ERG, ETS (erythroblast transformation-specific)-related gene; ERE, Estrogenresponse elements; STAT, Signal transducer and activator of transcription; NF-κB, Nuclear factor kappaB; PPAR-γ-Peroxisome proliferator-activated receptors-γ; FGF, Fibroblast growth factors; VEGF, Vascular endothelial growth factor; TGF, Transforming growth factor; TF, Tissue factor; CTGF, Connective-tissue growth factor; EGF, Epidermal growth factor; TNF, Tumor necrosis factor; IFN, Interferon; COX-2, Cyclooxygenase-2; MCP-1, Monocyte chemotactic protein-1; PARP, Poly (ADP-ribose) polymerase; FADD, Fas-associated protein with death domain; MAPK, Mitogen-activated protein kinase; EGFR, Epidermal growth factor receptor; ERK, Extracellular signal-regulated kinase; IL-1 RAK, Interleukin-1 receptor-associated kinase; PKA/B/C, Protein kinase A/B/C; JNK, c-Jun amino-Terminal kinase; IKK, IκB kinase; CXCR4,C-X-C chemokine receptor type 4; EGFR, Epidermal growth factor receptor; H2R, Histamine H2 receptor; LDL-R, Low-density lipoprotein receptor; ITPR, Inositol 1,4,5-triphosphate receptors; Bcl-xL B-cell lymphoma-extra large; cIAP, Cellular inhibitor of apoptosis protein; Bcl-2, B-cell lymphoma-2; PCNA, Proliferating cell nuclear antigen.

**Table 2:** The most recent clinical trials with curcumin.

Cancer	Drug	Title	Clinical Trial Number (NCT)	Trial Phase	Estimated Study Completion Date
**Breast**	Curcumin	A “Window Trial” on Curcumin for Invasive Breast Cancer Primary Tumors	NCT03980509	I	November, 2021
	Curcumin^®^ (CUC-01) with paclitaxel	Study of Efficacy of Curcumin in Combination With Chemotherapy in Patients With Advanced Breast Cancer	NCT03072992	II	July, 2018
**Prostate**	Curcumin	A Randomized, Double-Blind, Placebo-Controlled Trial of Curcumin to Prevent Progression of Biopsy Proven, Low-risk Localized Prostate Cancer Patients Undergoing Active Surveillance	NCT03769766	III	November, 2026
**Cervical and Uterine**	Immunomodulatory cocktail (Vitamin D, aspirin, Cyclophosphamide and Lansoprazole), pembrolizumab and Curcumin	A Phase II Investigation of Pembrolizumab (Keytruda) in Combination With Radiation and an Immune Modulatory Cocktail in Patients With Cervical and Uterine Cancer (PRIMMO Trial)	NCT03192059	II	June, 2022
